# Implementation determinants and strategies in integration of PrEP into maternal and child health and family planning services: experiences of frontline healthcare workers in Kenya

**DOI:** 10.3389/frph.2023.1205925

**Published:** 2023-09-20

**Authors:** Anjuli D. Wagner, Kristin Beima-Sofie, Mercy Awuor, Winnie Owade, Jillian Neary, Julia C. Dettinger, Jillian Pintye, Felix Abuna, Harison Lagat, Bryan J. Weiner, Pamela Kohler, John Kinuthia, Grace John-Stewart, Gabrielle O’Malley

**Affiliations:** ^1^ Department of Global Health, University of Washington, Seattle, WA, United States; ^2^ UW-Kenya, Nairobi, Kenya; ^3^ Department of Epidemiology, University of Washington, Seattle, WA, United States; ^4^ Department of Biobehavioral Nursing and Health Informatics, School of Nursing, University of Washington, Seattle, WA, United States; ^5^ Research & Programs, Kenyatta National Hospital, Nairobi, Kenya; ^6^ Department of Health Systems and Population Health, University of Washington, Seattle, WA, United States; ^7^ Department of Child, Family, and Population Health Nursing, University of Washington, Seattle, WA, United States; ^8^ Departments of Pediatrics and Medicine, University of Washington, Seattle, WA, United States

**Keywords:** pre-exposure prophylaxis (PrEP), pregnancy, postpartum, adolescent girls and young women (AGYW), implementation science, consolidated framework for implementation research (CFIR), implementation strategies

## Abstract

**Background:**

Delivery of PrEP to adolescent girls and young women (AGYW) and to pregnant women through maternal and child health (MCH) and family planning (FP) clinics is scaling up in Kenya. Evaluation of implementation challenges and strategies is critical to optimize delivery.

**Methods:**

We conducted focus group discussions (FGDs) with healthcare workers (HCWs) in MCH and FP clinics offering PrEP in a large implementation project in Kisumu, Kenya. Discussion guides were based on the Consolidated Framework for Implementation Research (CFIR). FGDs were audio recorded and transcribed. Directed content analysis was used to identify implementation challenges and strategies to overcome them.

**Results:**

Fifty HCWs from 26 facilities participated in 8 FGDs. HCWs believed PrEP integration was appropriate because it met the needs of AGYW and pregnant women by providing a female-controlled prevention strategy and aligned with policy priorities of elimination of vertical HIV transmission. They were universally accepting of PrEP provision, especially through MCH clinics, noting the relative advantage of this approach because it: (1) enabled high coverage, (2) harmonized PrEP and MCH visits, and (3) minimized stigma compared to PrEP offered through HIV care clinics. However, HCWs noted implementation challenges affecting feasibility and adoption including: (1) increased workload and documentation burden amid workforce shortages, (2) insufficient health care worker knowledge (3) multiple implementing partners with competing priorities (4) drug and documentation form stockouts. HCWs employed various implementation strategies to overcome challenges, including task shifting from nurses to HIV testing providers, patient flow modifications (e.g., fast-tracking PrEP clients to reduce wait times), PrEP demand generation and myth clarification during health talks, provider education, dedicated PrEP delivery rooms, and coordination with adolescent-friendly services. Additional suggested strategies to improve PrEP integration included community education to increase broader PrEP awareness and enable shorter counseling sessions, and task-shifting data entry and client risk assessments.

**Conclusions:**

HCWs were enthusiastic about the appropriateness and acceptability of integrating PrEP services into MCH and FP clinics but noted challenges to adoption and feasibility. Strategies to address challenges focused on improving provider time and space constraints, and increasing provider and client knowledge.

## Introduction

There has been continued progress in decreasing HIV incidence in sub-Saharan Africa over the past decade as a result of expanded treatment and increased use of pre-exposure prophylaxis (PrEP) (1–3). Despite these successes, there is still room for improvement, particularly in preventing HIV acquisition among adolescent girls and young women (AGYW) and eliminating vertical transmission. A disproportionate number of HIV infections in sub-Saharan Africa are occurring among AGYW—with an estimated 4,200 AGYW acquiring HIV each week in 2020 (4). An increasing proportion of vertical transmissions occur as a result of acute maternal HIV acquisition during pregnancy or lactation (5, 6).

PrEP is highly effective as a woman-controlled HIV prevention option (7), and is safe for use during pregnancy and breastfeeding (8–10). PrEP is recommended for populations with substantial risk of HIV acquisition, including AGYW and pregnant and postpartum people, by the Kenyan Ministry of Health and the World Health Organization (2, 11). Despite the benefits of PrEP and guidelines supporting PrEP use in these populations, major challenges remain at the individual, provider, and systems-level for ensuring PrEP is accessed, taken up, and appropriately continued by AGYW and pregnant/postpartum people most at risk of acquiring HIV (12, 13).

Utilizing existing clinical structures to reach pregnant people and AGYW may substantially expand PrEP uptake and adherence and reduce HIV acquisition in these populations. Delivering PrEP through integration with existing services such as Maternal Child Health (MCH) clinics and Family Planning (FP) clinics is promising (14–16). However, challenges to implementation and integration at the facility level exist (13, 17) and evaluation of implementation challenges and strategies within health systems are critical to inform future scale-up (17, 18). Specifically, understanding how best to approach integrating PrEP into busy clinics while ensuring appropriate HIV testing, adequate pre-initiation and adherence counseling, and minimizing impact on other critical clinic functions is essential to the success of PrEP programs in MCH and FP clinics (17).

We completed a large demonstration project, the PrEP Implementation for Young Women and Adolescents (PrIYA) project, which provided real-world programmatic delivery of PrEP via 37 MCH and FP clinics in Kisumu County, Kenya (15, 16). The PrIYA project screened >20,000 girls and women ≥15 years of age for HIV risk and offered PrEP counseling to all women, regardless of HIV risk. As part of a broad evaluation of this project, numerous barriers to PrEP uptake and continuation were identified. Community advisory board members noted community-level misconceptions that PrEP will make AGYW promiscuous, conflating PrEP with HIV treatment, and stigma and fear felt by AGYW accessing PrEP outside of a youth-friendly space (19). AGYW described misinformation related to cost, dosing, and focus populations for PrEP, misconceptions that were more pronounced among those receiving information from community outreach campaigns (20). AGYW also described fearing partner reactions and fearing that PrEP interfered with either contraception or fertility as barriers to using PrEP, even when at higher risk of HIV acquisition (21). In this specific study, we explored HCW perspectives on barriers to PrEP delivery and strategies for overcoming those barriers that can be empirically tested in future studies as programs seek to integrate PrEP into existing clinical services.

## Methods

### Study design

We conducted focus group discussions (FGD) with healthcare workers (HCWs) from MCH and FP clinics who offered PrEP as part of the PrIYA project. Within the PrIYA project, integrated delivery of PrEP included: integrated PrEP screening and counseling and integrated PrEP medication dispensing within the MCH or FP clinic. Between October and December 2018, eight FGDs were conducted with 50 purposively recruited HCWs experienced with PrEP delivery through the PrIYA project. Participants were recruited through study staff and in collaboration with facility leadership and were informed that their decision to participate in the FGDs would not impact their job. Half of the FGDs (27 HCWs) were conducted with PrIYA staff. PrIYA staff were full-time PrIYA employees who were tasked with working with diverse clinics to build sustainable systems for PrEP delivery within clinics and were responsible for PrEP delivery and implementation at 16 PrIYA project sites. The other FGDs (23 HCWs) were conducted with routine clinic staff working at the 21 newly expanded PrIYA-mentorship sites. These HCWs were full-time employees of the clinic who were trained by PrIYA team members to add PrEP delivery to their existing clinic activities. PrIYA project sites and PrIYA-mentorship sites were selected in collaboration with the Kisumu County Department of Health to maximize patient volumes and geographic locations.

### Ethical review

This study was reviewed and approved by the Kenyatta National Hospital/University of Nairobi Ethics and Research Committee and the University of Washington Institutional Review Board. Participants provided written informed consent for participation in focus group discussions.

### Data collection

Semi-structured topic guides were developed based on the Consolidated Framework for Implementation Research (CFIR), a flexible, meta-theoretical framework used to describe heterogeneity in implementation across settings, as well as the relative effect of key determinants in influencing implementation outcomes (22). FGDs explored determinants of early implementation acceptability, appropriateness feasibility, and adoption, and strategies that facilitated improved implementation. FGDs were conducted in English by two female Kenyan social scientists (MA, WO) who did not have prior relationships with the participants. One of five note takers (4 female, 1 male) was also present at all FGDs. FGD facilitators and note takers were trained on the goals of the study, the clinical effectiveness of PrEP, and the importance of maintaining participant confidentiality and neutrality. Participants were apprised of the purpose of the research through the consent form. FGDs were conducted, and audio recorded, in a quiet, confidential setting and lasted an average of 104 min. Facilitators wrote detailed FGD debrief memos (23), and transcription was ongoing throughout data collection.

### Data analysis

Directed content analysis (24) was used to identify the main CFIR constructs influencing HCW beliefs about PrEP delivery through MCH/FP clinics. All transcripts were coded using an iteratively developed codebook. The codebook was developed using a deductive approach, based on CFIR domains and constructs, and an inductive approach to identify implementation strategies. The coding team (KBS, ADW, GO) included qualitative and implementation science researchers with >10 years of experience working in HIV prevention in Kenya. Discussion by the coding team helped operationalize the CFIR constructs into codes and focused primarily on constructs within the CFIR inner setting, intervention characteristics, and process domains. Using open coding, an additional set of codes were developed to capture specific strategies used or identified to improve PrEP delivery, including strategies related to integration, logistics, education, counseling, uptake, adherence, and task shifting.

Dedoose was used to support data management and analysis (Dedoose version 7.0.23, Los Angeles, CA, USA: Sociocultural Research Consultants, LLC). Members of the coding team (KBS, ADW, GO) independently coded one-third of the transcripts using the final version of the codebook. Code application and text segmentation was then reviewed by a second member of the team and any disagreements were noted and resolved through group discussion. The team synthesized the coded data to identify key themes related to factors impacting PrEP implementation in MCH/FP clinics, as well as recommended strategies for improving PrEP implementation in these settings. The FGD facilitators were involved in the development of this manuscript to ensure findings reflect participant experiences shared during FGDs.

## Results

**Figure 1:**
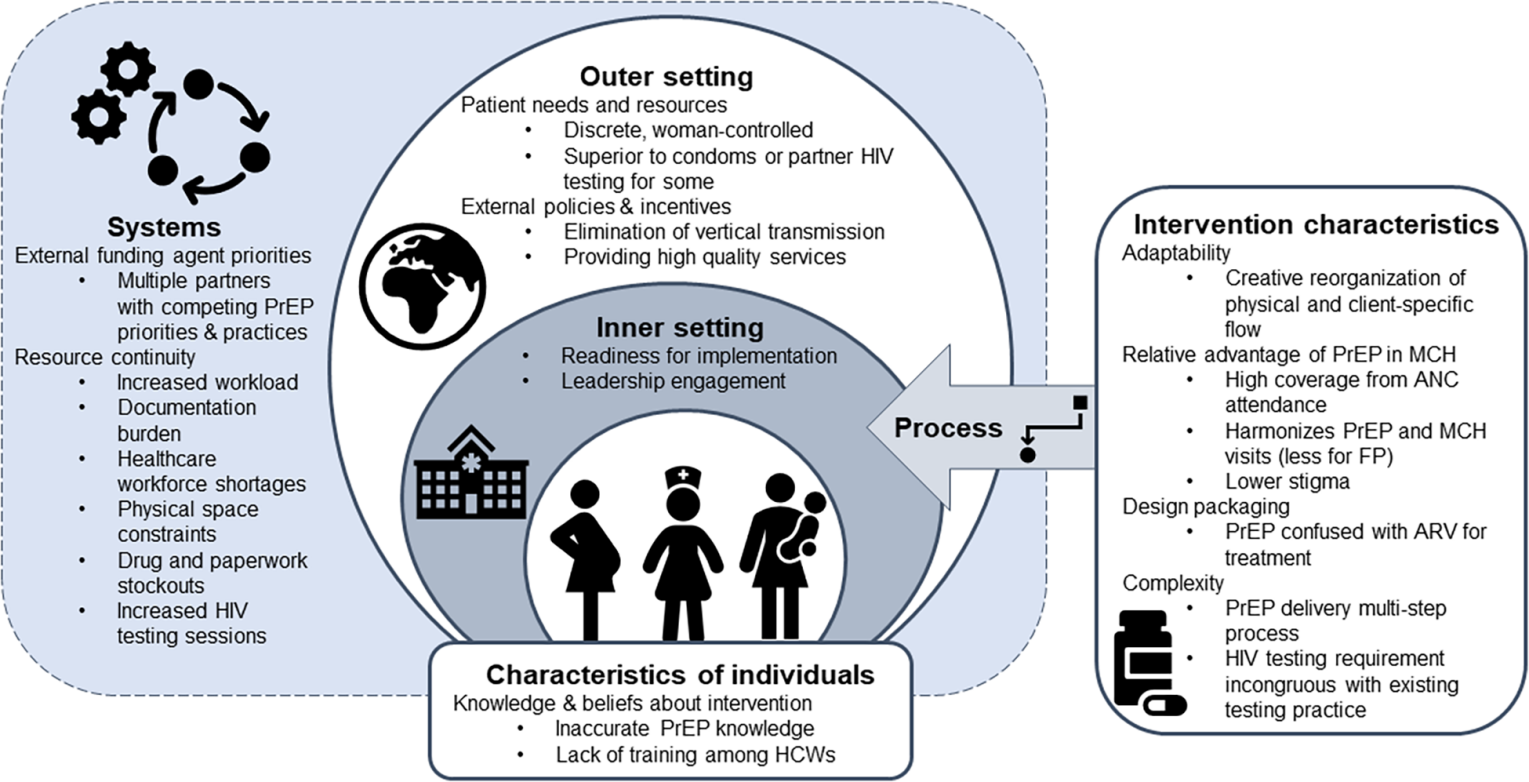
Overview of CFIR determinants affecting HCW perceptions of the acceptability, adoption, appropriateness and feasibility of delivering PrEP within MCH and FP services.

Fifty HCWs from 26 facilities participated in 8 FGDs. Demographics have been previously reported (25). The majority (72%) were female, and the median age was 28 (IQR: 26–32). HCWs were primarily nurses (56%), clinical officers (16%), and nurse counselors (12%). HCWs had a median of 13 months’ experience providing PrEP (IQR: 10–18) and 92% had received additional training on providing PrEP specifically to AGYW. PrIYA staff reported an average of 3 months more experience providing PrEP to AGYW when compared to HCWs from PrIYA mentorship sites. Overall, participants were enthusiastic about PrEP provision for AGYW and pregnant women via FP and MCH clinics, finding this integration strategy to be acceptable and appropriate. Despite high enthusiasm, HCWs described specific challenges to integration that limited feasibility and adoption. HCWs were able to overcome many barriers to PrEP integration through adapting delivery strategies to optimize implementation in their respective clinics. Grounded in the CFIR, we identified key determinants influencing HCW perceptions of acceptability, adoption, appropriateness, and feasibility, and potential implementation strategies for future integration (Figure 1).

### PrEP delivery through MCH/FP clinics addresses policy priorities, is easily adaptable, meets patient needs, and provides a relative advantage over existing delivery strategies (outer setting and intervention characteristics)

HCWs felt PrEP delivery through MCH/FP clinics aligned with policy priorities of elimination of mother-to-child HIV transmission, policy priorities to reduce HIV acquisition among young women, and their larger overarching mission (as HCWs) of providing high quality services to patients, relating to the outer setting domain.

*“One thing we have agreed is that introduction of PrEP at the MCH/FP has had more advantages than disadvantages, so it is upon us as the health workers who are at those various stations to carry on because the reason as to why we are here is to give quality service to our clients and all of us want to help in reduction of HIV prevalence in our country, so it is upon us to change our attitude and maybe not to wait for support supervision (laughter)…..it is upon us to embrace the new intervention that has come and give good services to our clients”—*29 year-old male, Mentorship clinic

HCWs found PrEP delivery to be acceptable because it could be easily adapted and optimized within their clinic setting, relating to the intervention characteristics domain. Facility-specific adaptations identified by HCWs included determining the best way to integrate PrEP into the physical- and client-specific flows in that facility.

*“We had the plan… but when we reached the facility we had to deliver PrEP according to the flow of how the facility works because not all facilities are the same, so we had to work with what we found in the facility…”*—45 year-old female, PrIYA nurse

Finally, HCWs universally believed that oral PrEP was appropriate because it met the needs of AGYW by providing a discrete, female-controlled prevention strategy, relating to the outer setting domain. HCWs were enthusiastic about PrEP provision to AGYW and pregnant women through MCH and FP clinics as it allowed them to accommodate the complex reproductive health counseling needs of AGYW patients. They frequently referenced MCH delivery as advantageous because it enabled high coverage, harmonized PrEP and MCH visits, and lowered stigma compared to PrEP offered through HIV care clinics. FP clinic provision was viewed as slightly less advantageous, because FP visits did not sync as well as with PrEP delivery visits, but still provided an access point with lowered stigma when compared to HIV care clinics.

*“These are women, adolescents and women of reproductive age. MCH will offer all those services that they need– family planning, child immunization, ANC (antenatal care) and all those things so they just come and they do all those things at one go.”—*24 year-old female, Mentorship clinic

*“(O)ne of the advantages I see myself through delivery of PREP through MCH is that there is no stigma associated with clients. When (they) go to the MCH they are very comfortable going there, they do not have any issues.”—*27 year-old female, PrIYA nurse

Importantly, although PrEP integration into MCH and FP clinics addressed many patient needs and had lower stigma than HIV care clinics, HIV stigma surrounding PrEP remained for some PrEP clients and confusion around PrEP as being an antiretroviral created challenges to PrEP retention and adherence, relating to the intervention characteristics domain. For example, HCWs noted one of the biggest challenges to initial uptake by women was related to the design quality and packaging. At the time of the study, PrEP was in an identical bottle to Truvada medication for HIV treatment, which led to confusion about why someone not living with HIV would take an antiretroviral (ARV), and fear of reactions by others who would assume the woman was living with HIV if she was seen with PrEP pills. These fears also affected retention and adherence among those who initially accepted.

*“…there are people who started PrEP but because they (heard), ‘hey! That drug is ARVs, used by people who are HIV positive,’ now they will stop at some point and now they will not come to the facility and probably they are not even at the facility that we serve.”—25 year-old female, PrIYA nurse*

### The biggest challenges to PrEP integration centered on readiness for implementation, especially available resources and intervention complexity

Despite noted advantages, integrating PrEP into MCH/FP clinics was not without challenges, particularly around integrating the requirements of PrEP delivery within already busy clinical settings. HCWs noted early phase implementation challenges affecting adoption, that mirror implementation of many new programs, including: (1) increased workload (including time for documentation, counseling, running lab tests) relating to the systems domain (2) lack of HCW knowledge and training, relating to the characteristics of individuals domain (3) multiple implementing partners with different PrEP priorities at the same site, relating to the systems domain and (4) drug and paperwork (e.g., paper registries and PrEP clinical monitoring tools) stockouts, relating to the systems domain.

Although enthusiastic, HCWs acknowledged that PrEP is a more complex intervention than most other HIV prevention options. As noted above, PrEP initiation requires clinical assessments, counseling, laboratory testing, side effect monitoring, and regular HIV testing, which results in increased time with clients, HCW workload, and training requirements for staff compared to other combination prevention strategies.

*“Providing PrEP is actually more technical than those other HIV preventing measures, because you need a lot of time… unlike other preventive measures, (like) condoms, you could only give (it) out to those who do not know (have experience) and being that it is something new, it takes a lot of time for a client to understand what you are talking about.”—*24 year-old female, PrIYA nurse

Understanding documentation and staffing requirements to implement PrEP in MCH and FP presented substantial barriers to early adoption and perceptions of feasibility of integrated PrEP delivery. HCWs struggled with staffing shortages, reporting that they were often “*only two nurses within the MCH/FP, and you are not there to offer PrEP only, you also offer other services so the workload sometimes becomes much” (28-year old male, PrIYA nurse),* or they were required to rotate through other departments while trying to keep up with their PrEP delivery responsibilities. Similarly, documentation presented a large burden in the beginning, and participants described challenges figuring out how to navigate the multiple PrEP-specific tracking documents.

*“There was a challenge because people were like, now we have been added more work and then the daily activity register was introduced… then there is PrEP register at the end of the month, nearly three reporting tools…”—*28 year-old female, Mentorship site

Sometimes this required harmonization across PrEP documentation in multiple locations or going to another clinic to complete their PrEP documentation. However, with time, experience, and improved efficiency with PrEP implementation, despite these initial challenges, HCWs reported “*later on we came to realize that the work actually is very minimal* (28 year-old female, Mentorship site)”.

The requirements for additional HIV testing caused confusion among facility staff who had not been sensitized on PrEP delivery. HCWs reported challenges with facility HIV testing services (HTS) counselors who were not aware of the repeat HIV testing requirements and would “*send her back (saying) that no, the client is not yet due for retest” (32-year old female, PrIYA nurse)*. HCWs also noted the challenges of delivering PrEP in MCH clinics when there were competing priorities from implementing partners, NGOs, and the Ministry of Health. They described added responsibilities without added staffing to provide services.

*“…we have so many NGOs bringing in a lot of activities in the MCH, so we would find (partner 1), brought (project 1) they have something they are bringing, (partner 2) want(s) to bring their own (project 2), so we had like very…very many things to do at a time and we were just here nursing. They bring the activities but they don’t bring their nurses, we are the ones to incorporate everything and we still had our things to do, so I think that was also a challenge.”—*26 year-old female, PrIYA nurse

In addition to available workforce, competing priorities and time, HCWs reported other challenges that limited feasibility, including procuring physical supplies, which was noted as being critical to ensuring facility buy-in and early support of PrEP delivery. National HIV guidelines for PrEP delivery recommend, but do not require, laboratory testing including creatinine clearance and Hepatitis B surface antigen tests when available. Ensuring facilities had adequate supply resources—including lab testing supplies, PrEP commodities, and PrEP documentation—was important for ensuring facility support of PrEP integration.

*“…I know that currently there are issues with PrEP drugs (stockouts), like we were instructed not to initiate new clients and just to maintain those who are already on PrEP…, this client is still at risk (and) has come for PrEP as a new client, will you give or will you not give and if you fail to give and it happens that this client seroconverts… PrEP stock is a big thing that they need to do, yes.”—*28 year-old male, Mentorship clinic

### Implementation facilitators and strategies that supported successful integration and high PrEP uptake included leadership engagement, open communication, and clinic flow optimization

Engagement with facility leadership was essential for overcoming challenges to PrEP adoption and feasibility, relating to the inner setting domain. In particular, the facility in-charge was critical in addressing and overcoming many of the early challenges to early adoption of PrEP including, space requirements for PrEP delivery, navigating conflicts between Ministry of Health staff, other partner organizations, and the PrIYA nurses, supporting PrIYA nurses in the case of supply challenges, and leading the integration of PrEP into facility activities as more than an external program.

*“In my facility we have worked as a team, and the team include(d) the MOH, (implementing partner) and us… we get our supplies from (implementing partner) pharmacy and we have never run out of stock, the matron in-charge at that time made work very easy for us.”-* 26 year-old male, PrIYA nurse

In addition to facility leadership, engagement and buy in from all cadres of facility staff was critical to ensuring smooth PrEP delivery in the facilities. Facility staff supported PrEP delivery through sharing responsibilities, educating clients on HIV prevention and PrEP, and sharing physical spaces as needed.

*“Our lead was really consulting with the HTS lead in our facility and I remember there was this one particular day we lacked the questionnaire and he said just give me one I go and do the photocopy. So he was really in the forefront just to make sure we are doing the screening…”—*43 year-old female, Mentorship clinic

**Figure 2:**
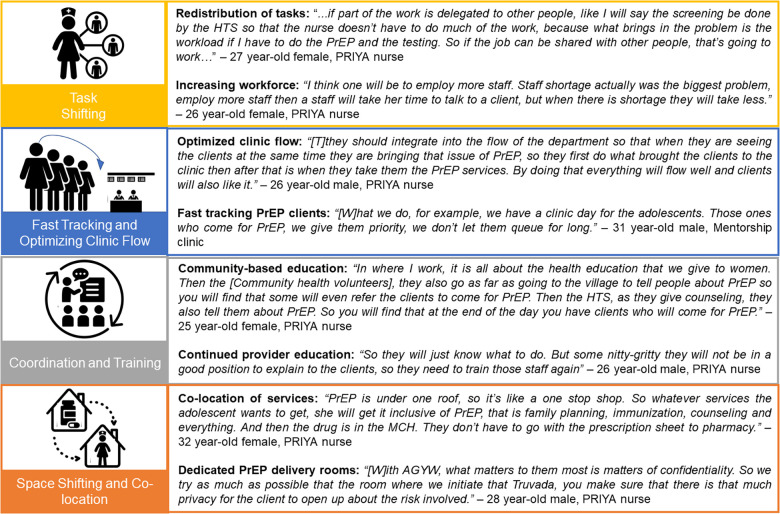
HCW identified strategies to integrated PrEP delivery.

In addition to engaging leadership, HCWs employed multiple implementation strategies, including: (1) task shifting, (2) fast-tracking and optimizing visit flow, (3) coordination and training of providers and clients in facilities and communities, and (4) space shifting and co-location (Figure 2). HCWs identified and organically tested a wide range of strategies, motivated to find context-specific solutions to deliver quality services.

*“(W)e, from different facilities, had to find something that would work in wherever we were working, because at the end of the day what will work for this facility might not work for the other, and we had to come up with ways to make PrEP delivery better for the future generation.”—*24 year-old female, PrIYA nurse

#### Strategy category 1: task shifting

Given the added time burden and complexity of PrEP delivery, some facilities identified ways to redistribute the added tasks of PrEP delivery across multiple HCW cadres. Through the flexible approach for PrEP delivery taken through the PrIYA project, each facility identified an optimal flow for clients in their clinic. Many facilities realized that HCWs working in HTS, in particular HIV testing counselors, were well equipped to conduct the HIV testing and HIV risk reduction counseling required for PrEP delivery.

“*(S)ome of the work was delegated to other departments like the HIV testing, and the (risk assessment) was relocated to the HTS to make the work of the nurse easier*”—27 year-old female, PrIYA nurse

Some providers described how increased patient volumes and amount of service time needed by PrEP clients could decrease the quality of information delivered, suggesting that task shifting could preserve quality of care. Specific suggested shifts included shifting screening, risk assessment, and counseling from nurses to HTS providers.

#### Strategy category 2: fast tracking and optimizing clinic flow

With multiple steps involved in PrEP delivery, some facilities described ways in which they modified clinic visit flow, integrating PrEP delivery into the cadence of visits both physically and conversationally. For example, since PrEP clients need to receive multiple sequential services within an already long MCH visit, providers recommended fast tracking or prioritization of PrEP clients within queues to save time, including at the laboratory or pharmacy.

*“At some point we give these clients first priority in terms of services and queue because they have extra services which is PrEP that they came for so if a client come for ANC then we’ll first prioritize on those who take PrEP.”—*26 year-old male, PrIYA nurse

HCWs also discussed optimizing PrEP visits by offering differentiated services to individuals with good PrEP adherence, including longer intervals between appointments and multi-month drug dispensing to better align with mother and infant services. In order to address documentation challenges, HCWs suggested assessing data sources for overlap, removing individual cards for HIV risk assessment or PrEP provision and instead relying on large multi-patient registers. Finally, some HCWs highlighted multiple competing copies of the same register housed at different clinics within the same facility, suggesting having either one master copy per facility or revised record numbering systems.

#### Strategy category 3: coordination and training

The complexity, for example the HIV testing requirements, and novelty of the PrEP intervention required initial education and sensitization of all facility staff in order to ensure their buy-in and support.

*“…as a facility we hold a meeting where all the health care providers at the facility, including everybody. We disseminated the same message to them. Then now we decided when to start with everybody having the knowledge of PrEP.”—*48 year-old female, Mentorship clinic

Developing strategies to train and retrain facility staff about PrEP was critical for ensuring clients were receiving accurate PrEP information and willing to come to the facility to access PrEP. For HCWs in the facility, ensuring they had information about PrEP in pregnancy, including “*adequate information, adequate (understanding of biology) of PrEP in relationship to pregnancy and the rest” (28 year-old female, PrIYA nurse)* was essential. Some pointed to the need to retrain providers to maintain a high level of technical competence. In addition to increasing technical knowledge, providers described broadly inclusive trainings as a way to increase provider buy-in for PrEP implementation.

*“Yes, most of the staff they accepted it because before it was rolled out the CME (continuing medical education meeting) that was conducted and the sensitization, almost all staff were involved so no one was left behind… they were aware about it, so we didn’t force some resistance.”—*28 year-old male, Mentorship clinic

HCWs also highlighted their important role of providing facility-based education for clients, often focused on myth-busting or providing factual information related to PrEP.

*“The myths on providing PrEP initially was too much, so you had to deal with the myths, you had to deal with the facts and then clients were so curious to know even if I myself was using PrEP, like why are you giving us PrEP, are you using it?”—*27 year-old female, PrIYA nurse

Beyond facility-based client education, HCWs pointed to the importance of multiple educational touch points from community to the facility; the purpose ranged from generating demand to introducing PrEP to providing specific details to facilitate decision-making. HCWs also noted the importance of peer education and peer leads to facilitate PrEP-related communication with adolescents.

#### Strategy category 4: space shifting and co-location

HCWs highlighted the importance of a dedicated PrEP delivery room within the MCH and FP clinics, which provided privacy, confidentiality, and minimized disruptions to other service areas. In addition, nearly all HCWs were in favor of co-locating PrEP delivery and dispensing activities within the MCH and FP clinics, rather than referring clients to HIV care clinics or sending them to fill prescriptions at a separate pharmacy building. One HCW described the ease that co-location provided for all.

*“…it is very easy to give PrEP at MCH because everything is integrated, so it will give the client and even the clinicians and the patient an easier time*.”—28 year-old female, Mentorship clinic

While some HCWs noted that there were logistical and coordination challenges in implementing co-location, especially dispensing medication outside of a pharmacy, co-location was generally felt to be worthwhile.

## Discussion

HCWs with experience delivering PrEP in MCH and FP clinics to AGYW and pregnant populations were enthusiastic about the acceptability and appropriateness of PrEP service integration but noted challenges to adoption and feasibility. Integration offered the benefits of leveraging high attendance at antenatal care services, a harmonized visit schedule between PrEP provision and antenatal care, less stigma from receiving care outside HIV care clinics, and alignment with policy priorities. Affecting perceived feasibility and adoption, HCWs felt integration increased workload and was affected by healthcare workforce shortages, physical space constraints, stockouts, multiple implementing partners with different priorities, complexity of PrEP-specific steps, and inaccurate PrEP knowledge or lack of training among HCWs. HCWs suggested strategies to improve PrEP integration within MCH/FP clinics, including task-shifting client risk assessments and other elements of visits including documentation, fast tracking at different areas, shifting the use of spaces for PrEP specific service delivery, and alternative communication tools and approaches for facility- and community-based education.

A 2020 systematic review of completed, ongoing, and planned implementation science studies, focused on PrEP delivery to pregnant and postpartum populations, noted several barriers at the levels of inner and outer setting, in addition to workload challenges (17). Noted determinants of adoption included whether guidelines specifically endorsed PrEP for pregnant populations, related to the outer setting. Determinants of implementation or fidelity included stockouts and provider knowledge, related to the inner setting. These results are similar to determinants identified in the present qualitative study. While there are numerous studies in the systematic review that assessed determinants of individual-level maintenance (e.g., demographic characteristics), or PrEP persistence, there were none that assessed determinants of sustained delivery at a clinic or provider level. A recent Kenyan study highlighted presence and gaps in availability of commodities and resources, identifying infrequent gaps in HIV and PrEP commodities (26). In the Kenyan context where the present study took place, the costs of PrEP drugs and lab tests are covered for patients in public health clinics.

Concerns have been raised by HCW in Zambian, Malawian, and Kenyan studies about the time constraints and workload associated with integrating PrEP services into MCH and FP (25, 27, 28). Indeed, PrEP related activities in PrIYA added 13 min (among PrEP non-initiators) and 18 min (among PrEP initiators) to their MCH/FP visits (13), representing additional service time that would be challenging to deliver by existing already overstretched HCW. However, integrating PrEP services into MCH and FP clinics has been successful in demonstration projects and in implementation studies, particularly those with additional staff provided. For example, in PrIYA uptake was 22% among pregnant and postpartum women and other AGYW (15, 16). In the PrIMA trial, which also involved additional staff support, PrEP acceptance was 18.6% among pregnant and postpartum women (29). In contrast, PrEP uptake was substantially lower in the PrEPARE implementation science study focused on MCH, which did not provide additional staff, at 3.9% among those offered PrEP (30) (*Sila & Wagner, under revision*). Similarly, after PrIYA staff departed, uptake of PrEP in FP clinics decreased to 4% (31). These four studies took place in Kenya; a systematic review noted that Kenya has been a leader in implementation research related to PrEP for pregnant and postpartum populations (17), with fewer studies planned or ongoing that measure uptake of PrEP outside of a trial setting. A study in South Africa observed substantially higher uptake of PrEP at 84% within a trial setting with additional staff; however, it was not possible to assess pre-trial enrollment attrition to determine whether the trial enrolled a population of women more likely to accept PrEP (32). A Ugandan cohort assessing pre-conception PrEP use among predominantly sero-different couples with fertility intention observed high PrEP uptake at 90%, but did not assess uptake during pregnancy (33). Future studies should consider how staffing ratios impact not only PrEP uptake, but also upstream steps of PrEP screening, counseling, and offer within busy MCH/FP clinics and test strategies to improve service provision reach broadly without adding new HCW. While time burden is one barrier to integrated PrEP delivery, provider training and knowledge, as well as retention, may be additional drivers of differential delivery.

Other studies have tested additional strategies to address a range of barriers similar to those we observed among HCWs in our study. One study utilized standardized patient actors to address lack of effective provider training and found that the training was associated with significantly improved counseling quality (34). The PrIMA study tested risk-guided versus universal offer of PrEP to assess whether a simplified PrEP offer was sufficient within routine practice to alleviate the time burden of PrEP-specific risk screening; this trial concluded that universal offer is superior to risk-guided offer due to its simplicity and comparable performance (29). Point-of-care sexually transmitted infection testing was assessed to address the barrier of low risk perception; this pilot found that PrEP uptake was significantly higher among women who accepted point-of-care sexually transmitted infection testing (35) and point-of-care testing was highly acceptable (36). Flow reorganization, task shifting, and provider training were tested to enhance the efficiency of integrated PrEP delivery within the PrIYA study (13). Within an ongoing South African stepped care trial, enhanced counseling and biofeedback plus rapid PrEP collection using HIV self-testing to expedite visits are being tested to decrease time and enhance continuation (37). Outside the context of pregnancy and postpartum, other studies have assessed the impact of an efficiency-focused “one stop shop” for PrEP delivery in a similar context, finding decreased waiting time, increase acceptability, and no changes in PrEP initiation and continuation (38).

Within the present qualitative study, numerous potential strategies were suggested to address barriers to PrEP delivery within MCH/FP clinics. In the context of limited resources, it is critical to prioritize which implementation strategies to adopt, ideally based on empiric testing. Prioritization methods within implementation science are evolving and being assessed for pragmatic utility (39, 40). Within the PrEPARE implementation science study, which is piloting implementation strategies to improve integrated PrEP delivery in MCH, the strategies identified in the present qualitative study were prioritized by HCWs and other key stakeholders using a series of quantitative surveys and ranking approaches (41). Prioritized strategies that were feasible to implement in the absence of additional staffing were then packaged for testing within MCH clinics in Kenya. Recent results for one implementation strategy package offering video education, HIV self-testing, and co-located PrEP dispensing, demonstrated significant improvements in PrEP screening, PrEP offer, PrEP knowledge, and client satisfaction. However, as mentioned above, PrEP acceptance among women offered PrEP was substantially lower than in the PrIYA study, suggesting that insufficient staffing is a major barrier to offering integrated PrEP (30) (*Sila & Wagner, under revision*). PrEPARE is currently testing two additional bundles of strategies to assess their impact on implementation outcomes for integrated PrEP delivery in MCH clinics in Kenya.

This study has several limitations. Most notably, half of the participants were staff members of a study focused on delivering integrated PrEP. Their roles focused on being ambassadors and implementers of PrEP; they may be more optimistic about the acceptability and feasibility of PrEP delivery and may not typify the usual staffing in public clinics. During the study period, there were substantial changes in the implementing partners who supported service provision, which impacted contracts for non-study staff; it is possible that the reflections from non-study staff reflect recent challenges with donor-imposed priority setting and lack of autonomy. Finally, these data were collected several years ago and the outer setting contextual factors captured during early implementation may differ from modern outer setting contextual factors.

## Conclusion

Overall, HCWs with experience delivering integrated PrEP in MCH and FP clinics to pregnant women and AGYW populations found this integration acceptable and appropriate. They highlighted that—for pregnant women—integration takes advantage of high attendance at antenatal care services and can align with visit schedules. For women in MCH and FP, delivery outside of an HIV care clinic was important to reduce stigma. Co-delivery of PrEP and MCH or FP services aligned with policy priorities of eliminating vertical transmission of HIV and providing comprehensive HIV prevention services. HCWs identified a range of barriers related to adoption and feasibility, including HIV testing and human resource shortages, documentation, stockouts, physical space constraints, complexity of PrEP delivery, and gaps in knowledge for providers and clients. Suggested implementation strategies, that improved adoption and perceived feasibility, included task shifting, fast tracking, communication aids and approaches, and shifting physical spaces should be further explored in future studies to better understand when and how to best employ these approaches.

## Data Availability

The raw data supporting the conclusions of this article will be made available by the authors, without undue reservation.
